# Effects of Lactic Acid Bacteria Fermentation on the Release and Biotransformation of Bound Phenolics in Ma Bamboo Shoots (*Dendrocalamus latiflorus Munro*)

**DOI:** 10.3390/foods14152573

**Published:** 2025-07-23

**Authors:** Liangshi Zhang, Anping Li, Hemei Liu, Qifeng Mo, Zhengchang Zhong

**Affiliations:** 1 School of Food Science and Engineering, Central South University of Forestry and Technology, Changsha 410004, China; zhangliangshi1117@163.com (L.Z.); liu19973548001@163.com (H.L.); 13250401636@163.com (Q.M.); 2 National Engineering Research Center of Rice and Byproduct Deep Processing, Central South University of Forestry and Technology, Changsha 410004, China; 3 College of Food Science, Tibet Agricultural and Animal Husbandry University, Nyingchi 860000, China

**Keywords:** lactic acid bacteria, fermentation, bamboo shoots, polyphenols

## Abstract

Lactic acid bacteria fermentation has the potential to enhance the biological activity of bamboo shoot polyphenols. The aim of this study was to investigate the release pattern and biotransformation mechanism of bound phenols from bamboo shoots prepared by fermentation with *Lactobacillus acidophilus*, *Pediococcus pentosaceus*, and *Lactobacillus plantarum*. The results showed that compared with unfermented controls, bound forms of vanillic acid, p-coumaric acid, and ferulic acid significantly decreased, while their free forms increased substantially after 6 d fermentation (*p* < 0.05). Quantitative analysis revealed particularly dramatic transformations for p-coumaric acid, which showed a 30–3000% increase in free form, and ferulic acid with a 203–359% increase in free form. *Pediococcus pentosaceus* demonstrated outstanding performance in bound phenol release and conversion, correlating with its higher β-glucosidase (0.67 U/g) and ferulic acid esterase (0.69 U/g) production. FITR, SEM, and IFM also demonstrated that LAB fermentation led to changes between free and bound phenols in bamboo shoots. These results demonstrate *Pediococcus pentosaceus* fermentation most effectively liberates bound phenolics, significantly improving their bioavailability for functional food applications.

## 1. Introduction

*Dendrocalamus latiflorus Munro*, commonly known as Ma bamboo shoot, is a plant of the *Poaceae* family in the genus *Phyllostachys*. Ma bamboo shoots are not only delicious and crispy but also rich in bioactive substances such as polyphenols, sterols, and dietary fiber, thus attracting increasing attention [1]. However, the harvesting period of bamboo shoots is concentrated, with strong respiration, easy fibrosis, and difficult storage, resulting in serious post-harvest losses [2].

LAB fermentation is a widely used method for processing fresh bamboo shoots. It not only extends the shelf life of bamboo shoots but also significantly enhances their flavor quality and bioactivity. Fermented bamboo shoots are very popular both at home and abroad [3]. LAB fermentation gives bamboo shoots a unique flavor, so there are many studies on the effects of LAB fermentation on the flavor and quality of bamboo shoots [4,5]. However, research on the impact of LAB fermentation on the polyphenol content in bamboo shoots is relatively scarce. This influence mechanism may be closely related to the release and transformation of phenols caused by the key enzymes secreted by LAB.

Polyphenols are the predominant active constituents in Ma bamboo shoots, most of which exist in bound states, and the proportion of free states is small [6]. These conjugated phenols are firmly attached to the cell wall matrix and other components, impeding their absorption in the gastrointestinal tract, and resulting in lower bioavailability than free phenols [7]. LAB fermentation of fruits and vegetables can enhance both the bioavailability and content of polyphenols while improving antioxidant activity [8]. For example, fermentation with *Lactobacillus plantarum* can increase the content of phenolic compounds and antioxidant activity in orange peel [9]. *Lactobacillus acidophilus* fermentation can significantly increase the total phenolic content and antioxidant capacity of kiwifruit flesh [10]. *Pediococcus pentosaceus* fermentation enhances both the bioaccessibility and antioxidant activity of polyphenols in blueberry puree [11]. This enhancement may be due to the release of bound phenols during fermentation, or to the role of LAB metabolites in polyphenol transformation. Certain LAB have been shown to produce β-glucosidase and ferulic acid esterase, thereby influencing polyphenol dynamics during fermentation [12,13].

Different LAB fermentations have different effects on polyphenols. Based on these documented effects of LAB fermentation on polyphenol liberation and transformation in various plant matrices [9,10,11], and considering that *Lactobacillus acidophilus*, *Pediococcus pentosaceus*, and *Lactobacillus plantarum* are the most commonly used commercial strains for bamboo shoot fermentation, the present study aims to investigate the effects of these three selected LAB strains on the polyphenol content and bioactivity of bamboo shoots. Through this investigation, we seek to elucidate the mechanism of polyphenol conversion during fermentation, thereby providing a theoretical basis for optimizing LAB fermentation to improve the bioactivity of bamboo shoots.

## 2. Material and Methods

### 2.1. Materials and Reagents

Fresh Ma bamboo shoots were purchased from Jieyang City, Guangdong Province, China. *Lactobacillus acidophilus* (CGMCC 26888), *Pediococcus pentosaceus* (CGMCC 27356), and *Lactobacillus plantarum* (CGMCC 26887) were obtained from Zhengzhou Baiyibao Biotechnology Co., Ltd.(Zhengzhou, China). The individual phenolic standards were procured from Sigma-Aldrich, St. Louis, MO, USA. The Trolox, 2,2-diphenyl-1-picrylhydrazyl (DPPH), Folin–Ciocalteu, and 2,2′-Azino-bis (3ethylbenzothiazoline-6-sulfonic acid) diammonium salt (ABTS) standards were procured from Shanghai McLean Biochemistry & Technology Co. (Shanghai, China). The β-glucosidase and ferulic acid esterase kits were obtained from Suzhou Gerace Biotechnology Co. (Suzhou, China).

### 2.2. Preparation of Fermented Bamboo Shoot Samples

Fresh Ma bamboo shoots weighing about 1 kg were selected, shelled, washed, and then cut into strips with a length and width of 4 cm × 2 cm × 1 cm, boiled in boiling water for 1 min, and drained. Then the bamboo shoots were added into the fermenter and submerged with salt solution with a mass fraction of 4%, then inoculated with *Lactobacillus acidophilus*, *Pediococcus pentosaceus* and *Lactobacillus plantarum* at 1 × 10^8^ CFU/100 mL, respectively, and fermented at room temperature, and the samples were taken at intervals of 2 days until the fermentation of 16 d. The bamboo shoots that had been fermented for 6 d were used for the subsequent study, as preliminary studies showed that phenolic compounds reached maximum release at 6 d of fermentation, accompanied by peak activities of β-glucosidase and ferulic acid esterase, whereas extended fermentation periods led to a gradual decrease in both free and bound phenolic contents. The unfermented bamboo shoots were used as the control, and they were named as FBSLA, FBSPP, FBSLP, and BS. Part of the samples were taken fresh for the determination, while part of the samples were lyophilised and pulverized through a 100 mesh sieve for the determination of the subsequent experiments.

### 2.3. Determination of Polyphenol Content

The polyphenol content of Ma bamboo shoots was determined by the Folin–Ciocalteu method with slight modification [14,15]. For free polyphenol extraction, a 0.5 g sample was taken and added to 80% (*v*/*v*) methanol solution and sonicated at 50 °C for 30 min. After that, the operation was repeated three times by centrifugation at 8000 rpm for 10 min to obtain the supernatant. The combined supernatants were concentrated under reduced pressure at 45 °C and reconstituted in 10 mL methanol for free polyphenol quantification. For bound polyphenol analysis, the above centrifuged precipitate was hydrolysed by adding 4 M NaOH, filling with nitrogen, and avoiding light at 25 °C for 4 h. After that, the supernatant was centrifuged at 8000 rpm for 10 min, and the supernatant was extracted by adding ethyl acetate (1:1 *v*/*v*), and the upper extract was taken, and the extract was evaporated to complete dryness under reduced pressure at 45 °C. Then, redissolved in 10 mL methanol for bound polyphenol measurement. The total phenol content was the sum of the free phenol content and the bound phenol content.

### 2.4. Determination of Polyphenol Fractions

The determination of each component of polyphenols from bamboo shoots was utilized by HPLC [15] (Shimadzu Inc., Kyoto, Japan) equipped with a diode array detector (DAD) set at 280 nm. Protocatechuic acid, p-hydroxybenzoic acid, vanillic acid, vanillin, p-coumaric acid, and ferulic acid were selected as the standards to be prepared as solutions and filtered by 0.45 μm membrane to establish the standard curve. The chromatographic column was Agile ZORBAX SB-C18 (250 mm × 4.6 mm, 5 μm). The mobile phase consisted of A is methanol and B is 0.1% formic acid. The elution gradient is as follows: 0–5 min 90% B, 5–18 min 80–65% B, 18–22 min 65–50% B, and 22–30 min 50–35% B.

### 2.5. Determination of pH and Total Acid (TA) Content

The pH of Ma bamboo shoots was determined by a pH meter. A 10 g sample of fresh bamboo shoots was taken, crushed, centrifuged at 10,000 rpm for 20 min and the supernatant was taken for the determination.

The TA content of bamboo shoots was determined by the national standard method (GB 12456-2021 [16]). Titration was performed with 0.05 M NaOH solution. The TA content was calculated by the formula:

(1) x = (c × v × k)/m

In the formula, x denotes the TA content (g/100 g); c refers to the concentration of the NaOH standard solution (mol/L); v represents the volume of NaOH used in titration (mL); k is the conversion factor for the acid, which is 0.09 for lactic acid; and m is the mass of the bamboo shoot sample (g).

### 2.6. Enzyme Activity Assay

The β-glucosidase enzyme (β-GC) activity of bamboo shoots was determined by the β-GC kit. The crude enzyme solution was obtained by taking 0.5 g of the sample and adding an extractant. Then the reaction reagents were added sequentially and incubated at 37 °C for 30 min, and the absorbance value was measured at 405 nm. The enzyme activity of β-GC was defined as the activity of catalyzing the production of 1 μM of PNP from PNPG per minute per g of sample at 37 °C.

The ferulic acid esterase (FAE) activity of bamboo shoots was determined by the FAE kit. The crude enzyme solution was obtained by taking 0.5 g of the sample and adding an extractant, followed by centrifugation to collect the supernatant. Then the reaction reagents were added sequentially and incubated at 40 °C for 30 min, and the absorbance of the supernatant was measured at 340 nm. FAE enzyme activity was defined as one unit of enzyme activity per gram of sample hydrolyzing 1 nM of the substrate, ferulic acid methyl ester, at 40 °C per minute.

### 2.7. Determination of Antioxidant Activity

#### 2.7.1. DPPH Radical Scavenging Assay

The DPPH scavenging activity of bamboo shoots polyphenols was determined by a microplate reader [17] (Molecular Devices, San Jose, CA, USA). 180 µL of 0.1 mM DPPH solution was taken and mixed well with 20 µL of the solution to be measured and reacted for 30 min at 25 °C protected from light. The absorbance was measured at 517 nm. The formula was calculated as follows:

(2) DPPH radical scavenging activity (%) = (1 − A_1_/A_0_) × 100%

where A_0_ represents the blank control group and A_1_ is the sample group. A standard curve was made with Trolox, and the final results were expressed as Trolox equivalents per gram dry weight (μmol TE/g DW).

#### 2.7.2. ABTS Radical Scavenging Assay

The ABTS scavenging activity of polyphenols in bamboo shoots was determined by UV-1900 spectrophotometry [18] (Aoyi Instruments Co., Ltd., Shanghai, China). The ABTS stock solution (7.4 mM ABTS, 2.6 mM K_2_S_2_O_2_) was diluted with methanol to give an absorbance of 0.7 ± 0.02 at 734 nm to form a working solution of ABTS. A total of 20 µL of the test solution was mixed with 2 mL of ABTS working solution and reacted for 6 min in the dark. At the end of the reaction, the absorbance was measured at 734 nm. The ABTS radical scavenging activity was calculated in a manner similar to that of the DPPH scavenging activity.

### 2.8. Fourier Transform Infrared Spectroscopy (FTIR)

Freeze-dried samples obtained after 6 days of fermentation were mixed with KBr at a ratio of 1:100, ground and crushed with an agate mortar and pressed into tablets, then placed on a silicon platform and scanned with an infrared spectrometer (IRTracer-100, Shimadzu, Kyoto, Japan). The samples were scanned 64 times at a resolution of 4 cm^−1^ in the region of 4000~400 cm^−1^ to obtain the infrared spectra of the samples.

### 2.9. Scanning Electron Microscope (SEM)

Freeze-dried samples obtained after 6 days of fermentation were coated with gold and then observed using an SEM (Regulus8230, Hitachi, Tokyo, Japan) at a magnification of ×30,000.

### 2.10. Inverted Fluorescence Microscope (IFM)

Freeze-dried samples obtained after 6-day fermentation underwent free polyphenol removal prior to IFM (Ti2-E, Nikon Co., Ltd., Tokyo, Japan) analysis, with an eyepiece of 10× and an objective of 40×. The excitation wavelength of blue light was 361–389 nm (DM 415 suppression filter), and the excitation wavelength of green light was 465–495 nm (DM 505 suppression filter).

### 2.11. Data Analysis

Statistical analysis was conducted using one-way ANOVA followed by Duncan’s multiple range test (SPSS 22.0, IBM, Armonk, NY, USA) to compare all experimental groups (BS, FBSLA, FBSPP, FBSLP) simultaneously. Results are presented as mean ± standard deviation (*n* = 3), with different superscript letters indicating statistically significant differences (*p* < 0.05) between groups. Figures and charts are created using Origin 2021 software.

## 3. Results and Discussion

### 3.1. Impact of LAB Fermentation on Polyphenol Content in Bamboo Shoots

**Figure 1:**
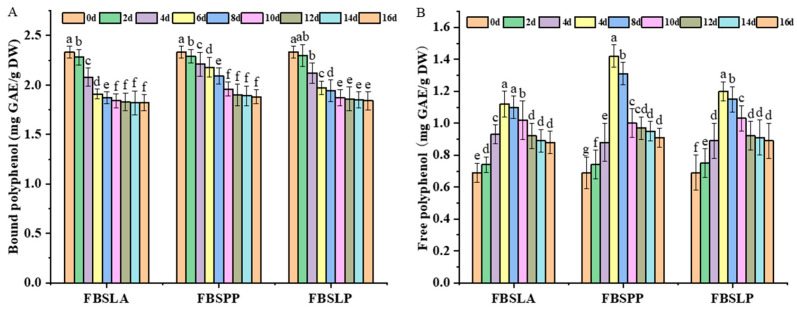
Changes in the content of bound phenols (**A**) and free phenols (**B**) in bamboo shoots during the fermentation process of LAB. The different letters on the bar chart indicate significant differences in the content of bound phenols and free phenols between the same sample and different fermentation times, with *p* < 0.05.

Using unfermented bamboo shoots BS as a control, the changes in the content of bound and free phenols contained in bamboo shoots during fermentation by different lactic acid bacteria are shown in Figure 1. The polyphenols in bamboo shoots were dominated by bound phenols. In the first 6 d of fermentation, compared with BS, the content of bound phenols in FBSLA, FBSPP, and FBSLP gradually decreased with the increase in fermentation time, while on the contrary, the content of free phenols gradually increased. From 6 d to 10 d of fermentation, both bound and free phenol contents of the three bamboo shoots decreased slowly with increasing time. From 10 d to 16 d, the contents of bound and free phenols in the three bamboo shoots gradually stabilized. This may be due to the rapid multiplication and vigorous metabolism of LAB in the first 6 d of fermentation, and the various enzymes and total acids produced by the fermentation contributed to the release of bound phenols, which are tightly bound to the plant cell wall, into free phenols [19]. The conversion of polyphenol release into free phenols was most pronounced on the 6th day of fermentation, so the sample from the 6th day was selected for subsequent experiments to explore the reasons for the changes in polyphenols due to LAB fermentation. At this time, the bound polyphenol content of FBSLA, FBSPP, and FBSLP was 1.91, 2.18, and 1.93 mg GAE/g DW, and the free polyphenol content was 1.12, 1.48, and 1.20 mg GAE/g DW, respectively. The ability of LAB fermentation to significantly increase the content of free polyphenols has also been confirmed in previous studies [20]. At the same time, free phenol properties are also unstable and susceptible to oxidative damage, so the free phenol content decreases in the later stages of fermentation.

### 3.2. Effect of LAB Fermentation on the Polyphenol Composition in Bamboo Shoots

**Figure 2:**
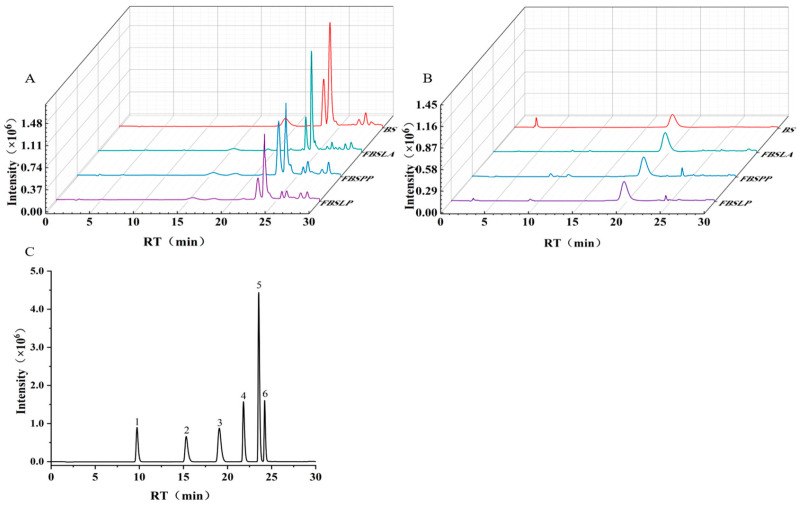
BPC diagram of polyphenols in bamboo shoots, bound polyphenols (**A**), free polyphenols (**B**) and standard (**C**). 1-Protocatechuic acid, 2-P-hydroxybenzoic acid, 3-Vanillic acid, 4-Vanillin, 5-P-coumaric acid, 6-Ferulic acid.

The composition of bound and free phenols contained in bamboo shoots after 6 d of LAB fermentation is shown in Table 1 and Figure 2. From the graphs, it can be seen that the fermentation by the three LAB had different effects on the content and composition of bound and free phenols in bamboo shoots.

Compared with the control BS, the fractions of vanillic acid, p-coumaric acid, and ferulic acid in the bound state were significantly reduced (*p* < 0.05), and those in the free state were significantly increased (*p* < 0.05) in the bamboo shoots fermented for 6 d. The reduction in bound vanillic acid was as high as 94%, 88%, and 88%, and the free state of ferulic acid was increased 202%, 359%, and 252% in FBSLA, FBSPP, and FBSLP, respectively, compared with the control BS. Ferulic acid is the most abundant phenolic acid in bamboo shoots, and after fermentation the bound ferulic acid decreased and the free ferulic acid increased, but the increase in free ferulic acid was much less than the release of bound ferulic acid. This suggests that LAB fermentation may not only contribute to the release of ferulic acid but also to its biotransformation.

Meanwhile, the contents of bound and free forms of p-hydroxybenzoic acid and vanillin were significantly increased (*p* < 0.05) in bamboo shoots after 6 d of fermentation, with the increase in bound p-hydroxybenzoic acid as high as 327%, 357%, and 285% in FBSLA, FBSPP, and FBSLP, respectively. In addition, the bound state protocatechuic acid was not detected in the fermented bamboo shoots, while the free state was detected. All indications are that not only the release of bound phenols but also the biotransformation among polyphenols occurred during the fermentation process of LAB. Previous studies have also shown that LAB fermentation can cause the corresponding transformation of plant polyphenols [21].

### 3.3. Biotransformation Among Polyphenol Components in Fermented Bamboo Shoots

**Figure 3:**
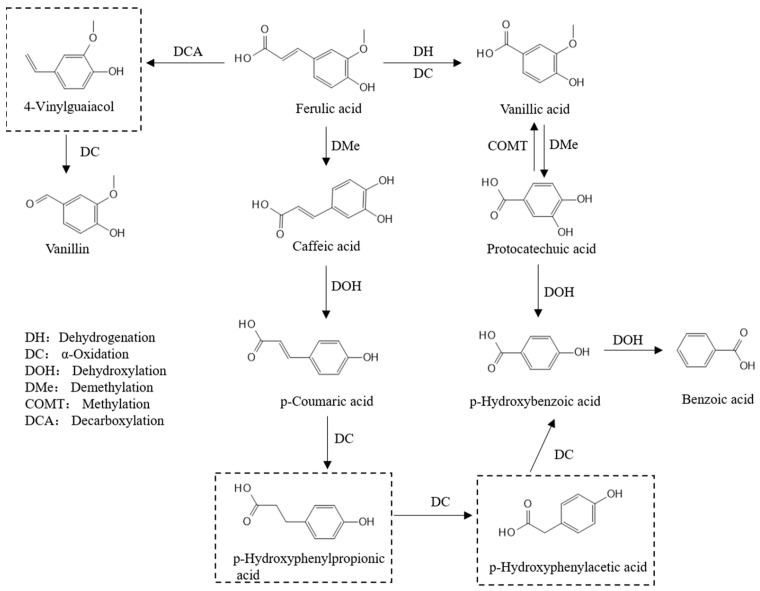
Conversion pathways of phenolic components in bamboo shoots fermented by LAB (compounds within dashed boxes represent undetected metabolites).

Based on Table 1, it can be deduced that bamboo shoots undergo biotransformation between their polyphenol fractions after fermentation by LAB. The transformation between polyphenols may occur through various pathways under the action of hydrolytic enzymes produced by microorganisms. The release of ferulic acid contained in bamboo shoots from the bound state to the free state after fermentation by LAB, but the two quantities were not equal, suggesting that ferulic acid may have undergone biotransformation. By searching the related literature, it was hypothesized that ferulic acid might be transformed through the pathways of benzoic acid and hydroxycinnamic acid [22,23], as shown in Figure 3. Ferulic acid can be converted to vanillin through decarboxylation and α-oxidation [24] and to vanillic acid through dehydrogenation and α-oxidation [25].

In the benzoic acid pathway, the corresponding conversions can occur between vanillic acid, protocatechuic acid, p-hydroxybenzoic acid, and benzoic acid [26]. Ferulic acid, caffeic acid, and p-coumaric acid are in the hydroxycinnamic acid conversion pathway; demethylation of ferulic acid can be converted to caffeic acid, and dehydroxylation of caffeic acid can be converted to p-coumaric acid [27]. P-coumaric acid can be converted to p-hydroxybenzoic acid through a series of α-oxidations, thus entering the benzoic acid pathway [28]. LAB may promote the release and transformation of phenolic compounds through processes such as depolymerization, hydrolysis, decarboxylase-mediated metabolism, and reductase activity [29].

The reduced amount of p-coumaric acid in the bound state was much larger than the newly added free p-coumaric acid in FBSLA and FBSLP for 6 d of fermentation, probably because it entered the metabolic pathway of benzoic acid and produced other related compounds. The contents of protocatechuic acid, vanillin, p-hydroxybenzoic acid, and p-coumaric acid were significantly higher in FBSPP than in FBSLA and FBSLP, both in the bound state and in the free state, suggesting that the fermentation by *Pediococcus pentosaceus* was more effective in the conversion and retention of phenolics in bamboo shoots.

### 3.4. Effects of LAB Fermentation on pH, TA, and Enzyme Activity in Bamboo Shoots

**Figure 4:**
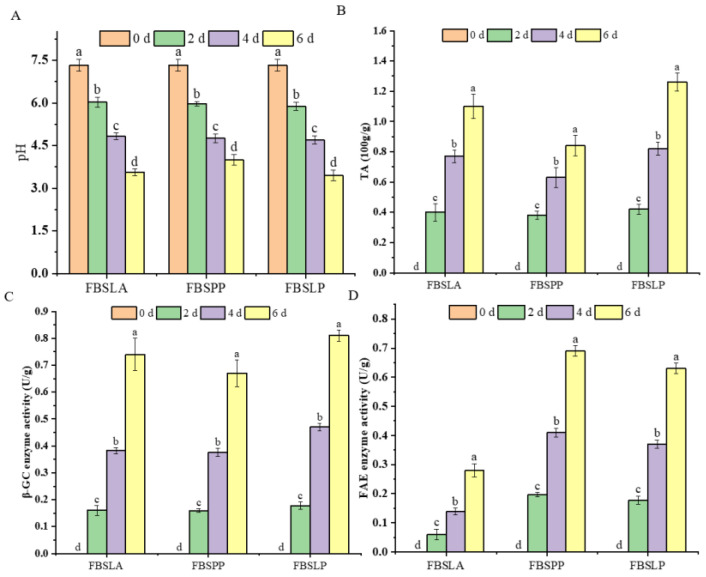
Changes in (**A**) pH, (**B**) TA, (**C**) β-GC enzyme activity, and (**D**) FAE enzyme activity in bamboo shoots prepared by LAB fermentation for 6 days. Different letters indicate significant differences in pH value, TA, and enzyme activity content for the same sample at different fermentation times, *p* < 0.05.

Figure 4 shows the changes in pH, TA, β-GC enzyme activity, and FAE enzyme activity in bamboo shoots prepared by LAB fermentation for 6 d. pH and TA can reflect the growth status of the strains and the degree of fermentation to some extent. Compared with BS, the pH of FBSLA, FBSPP, and FBSLP fermented for 6 d decreased from 7.33 to 3.56, 4.00, and 3.45, respectively, and the total acid increased from 0 to 1.10, 0.84, and 1.26 (100 g/g). This indicates that bamboo shoots are a good substrate for the growth and metabolism of LAB, and all three LAB were able to metabolize the sugars of bamboo shoots to produce organic acids.

**Figure 5:**
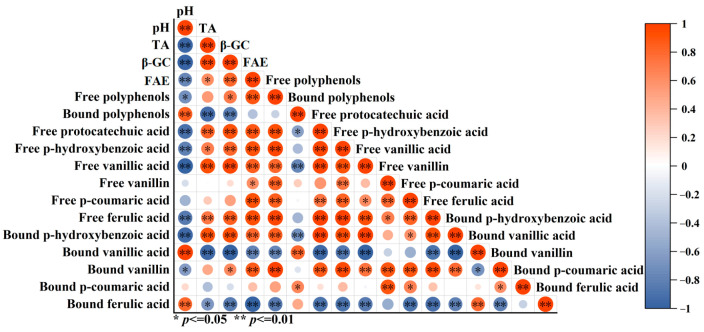
Correlation of pH, TA, β-glucosidase enzyme activity and polyphenol changes in bamboo shoots fermented by LAB. Red represents a positive correlation, blue represents a negative correlation. * indicates significant correlation, *p* < 0.05; ** indicates highly significant correlation, *p* < 0.01.

LAB fermentation not only produces acid but also produces a variety of enzymes such as β-GC and FAE, which are capable of hydrolyzing glycosidic and ester bonds, leading to the release of bound phenols. The correlation of pH, TA, β-GC enzyme activity, and FAE enzyme activity with the change in polyphenol content in bamboo shoots fermented by LAB for 6 d is shown in Figure 5. pH was significantly positively correlated with bound phenol content (*p* < 0.05, *R* = 0.85), and TA was significantly negatively correlated with bound phenol content (*p* < 0.05, *R* = −0.93), which indicated that the acidic environment contributes to the release of bound phenols. Similar findings have been reported in previous studies, which found that reducing the pH value from 6.00 to 3.00 can increase the extraction rate of apple polyphenols by nearly 48%, thereby promoting the release of apple polyphenols [30].

β-GC is a cellulose hydrolase capable of hydrolyzing glycosidic bonds and converting glycosidic polyphenols to free forms [31]. Figure 5 shows that β-GC enzyme activity showed a significant positive correlation with free phenol content (*p* < 0.05, *R* = 0.70) and a significant negative correlation with bound phenol content (*p* < 0.05, *R* = −0.84). In particular, the correlation coefficients, *R*, of β-GC enzyme activity with free and bound vanillic acid content reached 0.98 and −0.98, respectively. The results of Zhang et al. [32] similarly confirmed that the β-GC production capacity of LAB was closely related to the reduction in conjugated phenolic compounds and the increase in free phenols in fermented soya milk. FAE is able to hydrolyze the ester bonds between ferulic acid and polysaccharides such as cellulose and lignin in the plant cell wall, thereby releasing ferulic acid [33]. The correlation in Figure 5 showed that FAE enzyme activity was significantly and positively correlated with the free state ferulic acid content (*p* < 0.05, *R* = 0.84) and significantly and negatively correlated with the bound state ferulic acid content (*p* < 0.05, *R* = −0.83). This provides support for the conversion of bound ferulic acid to free ferulic acid in bamboo shoots.

The three LAB have different acid and enzyme production capacities and have different effects on the content and composition of polyphenols. The lower the pH of fermented bamboo shoots and the higher the enzyme activities of β-GC and FAE, the more bound polyphenols were released. Figure 1 shows that FBSLA and FBSLP have lower bound polyphenol content than FBSPP, indicating that it releases more bound polyphenols, but its free phenol content is still lower than that of FBSPP. This may be due to the fact that the polyphenols released by FBSLA and FBSLP were converted into other substances [34] or the fermentation environment of FBSLA and FBSLP caused polyphenol degradation. The release of polyphenols during fermentation cannot be achieved without the action of various enzymes involved in plant cell wall degradation. Different LAB produce enzymes differently. *Pediococcus pentosaceus* fermentation showed better results than *Lactobacillus acidophilus* fermentation and *Lactobacillus plantarum* fermentation for the release and conversion of bound phenols.

### 3.5. Comparative Antioxidant Activity of Free and Bound Phenol Extracts in Fermented Bamboo Shoots

**Figure 6:**
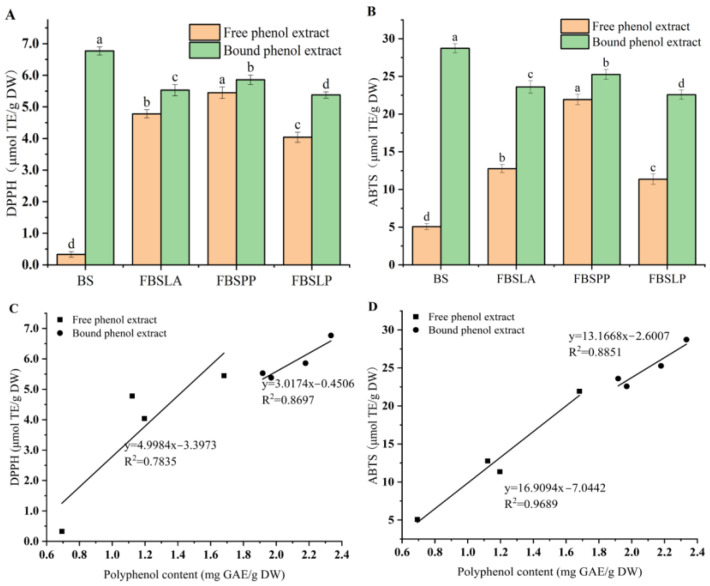
DPPH radical scavenging activity (**A**) and ABTS radical scavenging activity (**B**) of free phenol and bound phenol extracts from fermented bamboo shoots. Correlation between DPPH radical scavenging activity and free phenol and bound phenol content (**C**), and correlation between ABTS radical scavenging activity and free phenol and bound phenol content (**D**). The different letters in the bar chart indicate significant differences in the DPPH and ABTS free radical scavenging abilities between different samples, with *p* < 0.05.

The results of the DPPH and ABTS radical scavenging capacities of free and bound phenol extracts from bamboo shoots prepared by LAB for 6 d, and their correlation with the contents of free and bound polyphenols are shown in Figure 6. As can be seen from Figure 6A,B, the DPPH and ABTS radical scavenging capacities of the extracts of the four samples showed the same trend, with the DPPH and ABTS radical scavenging capacities of the free phenol extracts being, in descending order, FBSPP, FBSLA, FBSLP, and BS, and those of the bound polyphenols being, in descending order, BS, FBSPP, FBSLA, and FBSLP. This is consistent with the changes in the release of bound phenols into free phenols shown in Table 1. Similar observations were also reported when studying the effects of LAB fermentation on changes in phenolic compounds and antioxidant capacity of edible fungi [18].

Figure 6C,D showed that both free and bound phenol contents were significantly and positively correlated with ABTS and DPPH radical scavenging activities (*p* < 0.05), with correlation coefficients, *R*, of 0.885 and 0.933, and 0.984 and 0.941, respectively. Among the three fermented bamboo shoots, the free phenol extracts of FBSPP showed greater DPPH and ABTS radical scavenging capacities of 5.45 and 21.94 μmol TE/g DW, which was attributed to the greater release and conversion of bound phenols to free phenols in FBSPP.

### 3.6. FTIR Analysis of Bamboo Shoots Samples Before and After LAB Fermentation

**Figure 7:**
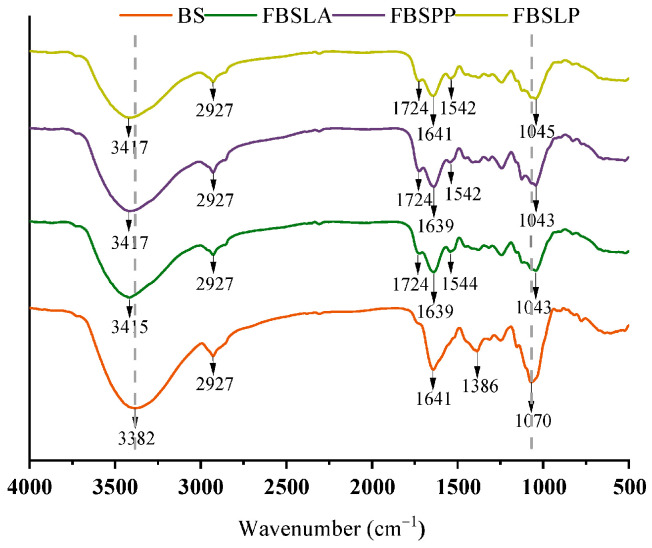
FTIR spectra of bamboo shoots before and after LAB fermentation (The dashed grey lines represent reference markers at 3382 and 1070 cm⁻¹ wavenumbers).

FTIR spectroscopy provides detailed information on the characteristics of chemical or biochemical substances present in a sample by analyzing the molecular vibrations of functional groups, including the stretching, bending, and twisting of chemical bonds. The FTIR spectra of bamboo shoots before and after fermentation by LAB are shown in Figure 7.

Fermented bamboo shoots, which are rich in dietary fiber, have a broad and strong absorption peak near 3382 cm^−1^, which corresponds to the stretching and bending vibrations of the -OH group in cellulose and hemicellulose where intermolecular hydrogen bonds are linked to the dietary fiber chains [35]. The absorption peak at 3382 cm^−1^ of the three bamboo shoots after LAB fermentation was blueshifted, and the intensity of the peak was reduced compared to BS. This indicates the breaking of intermolecular hydrogen bonds in some of the cellulose and hemicellulose. The breaking of hydrogen bonds weakens the interaction force between cellulose or hemicellulose and polyphenols and may contribute to the freeing of polyphenols from the cellulose or hemicellulose surface [36]. As shown in the results of the previous study, this leads to a decrease in bound phenols and an increase in free phenols.

The absorption peak at 1724 cm^−1^ usually corresponds to the C=O stretching vibration in esters and carboxylic acids [37]. The intensity of the absorption peak here increased after fermentation, suggesting a possible increase in carboxylic acid polyphenols, such as p-hydroxybenzoic acid content, during fermentation.

The absorption peak at 1641 cm^−1^, typically linked to C=C stretching vibrations in aromatic rings [38], is attenuated, likely due to changes in polyphenols during fermentation, such as dehydroxylation, which weakens the conjugation between phenolic hydroxyl groups and aromatic rings, thus affecting the C=C stretching vibration frequency. The peak at 1544 cm^−1^ is generally associated with the amide II band or N-H bending vibrations in aromatic amines [39]. The increased intensity of this peak post-fermentation may indicate the replacement of hydroxyl or carboxyl groups in polyphenolic compounds with amino groups or the formation of new nitrogen-containing compounds due to dehydrogenation reactions. The disappearance of the peak at 1386 cm^−1^, typically corresponding to O-H bending vibrations or C-H deformation vibrations in phenolic compounds [40], suggests that dehydroxylation of phenolic substances occurred during fermentation, leading to a reduction or loss of this group. The absorption peak at 1070 cm^−1^ is usually related to C-O stretching vibrations or the disruption of ether bonds [36]. Compared to the control, the fermented samples show significant shifts and reductions in peak intensity at this wavelength. Since compounds such as ferulic acid are bound to lignin via ether bonds [41], changes in this peak suggest that fermentation may lead to the cleavage of ether bonds, resulting in the release of bound polyphenols.

### 3.7. SEM Analysis of Bamboo Shoot Samples Before and After LAB Fermentation

**Figure 8:**
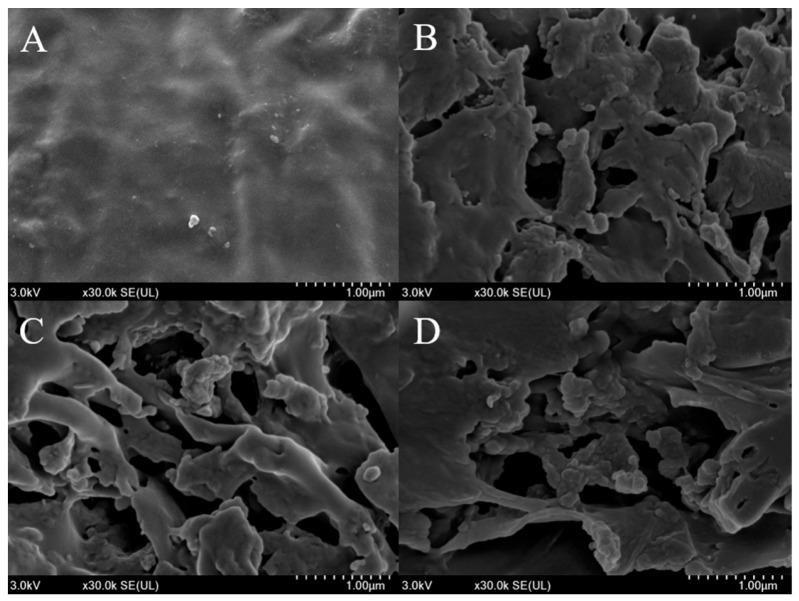
SEM of bamboo shoot samples before and after LAB fermentation: (**A**) BS; (**B**) FBSLA; (**C**) FBSPP; (**D**) FBSLP.

SEM observations of bamboo shoot samples before and after lactobacillus fermentation are shown in Figure 8. The surface of unfermented BS was smooth and flat, but the appearance of all three bamboo shoots fermented by LAB for 6 d showed holes of different sizes. This suggests that LAB fermentation is destructive to the cell walls of bamboo shoots and their compact structure. In addition, the increase in the number of pores in fermented bamboo shoots increases the specific surface area, the contact area with solvents such as organic acids, and the bound polyphenols are then more easily released and transformed [27].

β-GC has a certain ability to hydrolyse glycosidic bonds, leading to the disintegration of the cell wall structure. β-GC may be responsible for the obvious structural deletion that can be observed in FBSLP, FBSPP, and FBSLA. In addition, FAE can also disrupt the cross-linking structure in the cell wall, which makes the structural integrity of the cell wall weakened and leads to the formation of structural pores on the surface of bamboo shoots. The surface of FBSPP had more pores than FBSLA and FBSLP, which may be due to the fact that FBSPP produced more FAE.

### 3.8. IFM of Bamboo Shoot Samples Before and After LAB Fermentation

**Figure 9:**
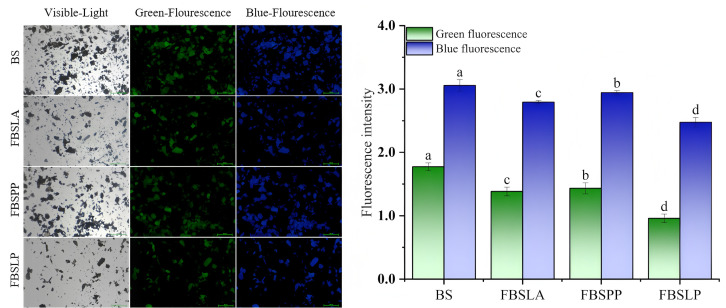
IFM observation of bamboo shoots before and after LAB fermentation (scale bar = 100 µm). The green and blue colors in the figure represent fluorescence emitted by phenolic compounds. The different letters in the bar chart indicate significant differences in green fluorescence and blue fluorescence intensities between different samples, with p < 0.05.

After the experimental samples were treated by removing the free polyphenols, all the binding phenols with strong autofluorescence, such as ferulic acid, were observed by fluorescence microscopy, which could reflect the changes in the binding phenols more accurately. The IFM observations of the samples fermented by LAB for 6 d are shown in Figure 9.

As can be seen from Figure 9, the green and blue fluorescence intensities of FBALA, FBSPP, and FBSLP were significantly weakened (*p* < 0.05) when obtained at 6 d of fermentation compared with BS. Among the three LAB fermented bamboo shoots, the green and blue fluorescence intensities were strongest for FBSPP, followed by FBSLA, and weakest for FBSLP. A significant correlation between fluorescence intensity and polyphenol content has been demonstrated [42]. The changes in green fluorescence and blue fluorescence intensity in Figure 9 were consistent with the changes in bound polyphenol content in Table 1. This suggests that the bound polyphenols were partially released from bamboo shoots after fermentation by LAB.

## 4. Conclusions

Bamboo shoots contain both bound and free phenols, of which bound phenols are predominant. *Lactobacillus acidophilus*, *Pediococcus pentosaceus*, and *Lactobacillus plantarum* not only released bound phenols but also biotransformed polyphenols during bamboo shoot fermentation. Compared with the control BS, the fractions of vanillic acid, p-coumaric acid, and ferulic acid in the bound state were significantly reduced (*p* < 0.05), and those in the free state were significantly increased (*p* < 0.05) in bamboo shoots fermented for 6 d. The free ferulic acid in the FBSLA, FBSPP, and FBSLP was increased by 202%, 359%, and 252%, respectively. TA, β-GC enzyme activity, and FAE enzyme activity produced by LAB fermentation were significantly correlated with the release and conversion of bound phenols (*p* < 0.05). FTIR, SEM, and IFM also demonstrated that LAB fermentation could lead to changes between free and bound phenols. Compared with FBALA and FBSLP, the free phenol content was higher in FBSPP, which means that *Pediococcus pentosaceus* was able to release and convert more bound polyphenols into free phenols, thus increasing the bioaccessibility of polyphenols in fermented bamboo shoots. Additionally, the enhanced phenolic bioavailability and functional properties of LAB-fermented bamboo shoots suggest promising applications as functional food ingredients, prebiotic supplements, or natural preservatives in the food industry. Furthermore, this approach aligns with sustainable food production by reducing post-harvest waste and creating value-added products from underutilized biomass.

## Figures and Tables

**Table 1:** Main types and contents of free and bound polyphenols (µg/g).

	Phenolic Content	BS	FBSLA	FBSPP	FBSLP
Bound	Protocatechuic acid	ND	ND	ND	ND
	p-Hydroxybenzoic acid	23.84 ± 0.94 ^d^	101.80 ± 4.68 ^b^	108.96 ± 5.86 ^a^	91.79 ± 3.16 ^c^
	Vanillic acid	66.75 ± 7.15 ^a^	4.30 ± 0.14 ^d^	8.03 ± 0.22 ^b^	7.98 ± 0.18 ^c^
	Vanillin	8.78 ± 0.23 ^d^	13.05 ± 0.16 ^c^	21.43 ± 2.23 ^a^	18.12 ± 0.08 ^b^
	p-Coumaric acid	173.52 ± 5.79 ^b^	136.32 ± 8.07 ^d^	208.28 ± 9.42 ^a^	165.58 ± 8.89 ^c^
	Ferulic acid	504.01 ± 10.20 ^a^	458.93 ± 9.26 ^b^	404.20 ± 6.94 ^c^	400.25 ± 7.77 ^c^
Free	Protocatechuic acid	0.01 ± 0.00 ^d^	23.52 ± 1.06 ^c^	32.26 ± 1.57 ^a^	26.78 ± 1.12 ^b^
	p-Hydroxybenzoic acid	ND	3.95 ± 0.08 ^c^	7.11 ± 0.06 ^a^	6.44 ± 0.08 ^b^
	Vanillic acid	83.21 ± 3.29 ^c^	107.13 ± 9.26 ^b^	119.57 ± 8.69 ^a^	118.17 ± 9.23 ^a^
	Vanillin	0.72 ± 0.04 ^c^	0.64 ± 0.07 ^d^	2.21 ± 0.07 ^a^	0.76 ± 0.12 ^b^
	p-Coumaric acid	0.67 ± 0.07 ^d^	0.87 ± 0.07 ^c^	20.83 ± 0.08 ^a^	14.36 ± 0.17 ^b^
	Ferulic acid	7.33 ± 0.20 ^d^	22.19 ± 0.62 ^c^	33.63 ± 1.13 ^a^	25.82 ± 1.46 ^b^

Note: ND indicates not detected; different letters (a, b, c, d) denote significant differences (*p* < 0.05) in the content of the same polyphenol component among different samples.

## Data Availability

The original contributions presented in this study are included in the article. Further inquiries can be directed to the corresponding authors.
